# Lossless Reconstruction of Convolutional Neural Network for Channel-Based Network Pruning

**DOI:** 10.3390/s23042102

**Published:** 2023-02-13

**Authors:** Donghyeon Lee, Eunho Lee, Youngbae Hwang

**Affiliations:** Department of Control and Robot Engineering, Chungbuk National University, Cheongju 28644, Republic of Korea

**Keywords:** network pruning, lossless reconstruction, convolutional neural network

## Abstract

Network pruning reduces the number of parameters and computational costs of convolutional neural networks while maintaining high performance. Although existing pruning methods have achieved excellent results, they do not consider reconstruction after pruning in order to apply the network to actual devices. This study proposes a reconstruction process for channel-based network pruning. For lossless reconstruction, we focus on three components of the network: the residual block, skip connection, and convolution layer. Union operation and index alignment are applied to the residual block and skip connection, respectively. Furthermore, we reconstruct a compressed convolution layer by considering batch normalization. We apply our method to existing channel-based pruning methods for downstream tasks such as image classification, object detection, and semantic segmentation. Experimental results show that compressing a large model has a 1.93% higher accuracy in image classification, 2.2 higher mean Intersection over Union (mIoU) in semantic segmentation, and 0.054 higher mean Average Precision (mAP) in object detection than well-designed small models. Moreover, we demonstrate that our method can reduce the actual latency by 8.15× and 5.29× on Raspberry Pi and Jetson Nano, respectively.

## 1. Introduction

Deep neural networks (DNNs) have been used in computer vision tasks with significant achievements, such as image classification [1,2,3], object detection [4,5,6,7,8], and semantic segmentation [9,10,11]. They are utilized in natural language processing and forecasting as well [12]. Generally, as the performance of DNNs improves, the number of parameters and computational complexity increase to deal with greater diversity from various databases. However, the trade-off between performance and cost renders the application of DNNs to resource-limited devices challenging.

To mitigate this trade-off, attempts have been made to design networks with high performance and efficiency. ResNet [2] constructs a deeper network based on a residual block. Although it achieves good results in image classification, it is computationally expensive because of the deeper structure. To apply this network on devices with different resources, various depth models were provided. In object detection, YOLO [6] introduces a one-stage regression method to enable real-time operations. Nevertheless, owing to the requirement for high computation, this method cannot be easily applied to resource-limited devices. Therefore, a smaller model termed tiny-YOLO was designed as well. However, these methods require delicate manipulation to configure the network and are inflexible in meeting the costs of various target devices.

Several studies have sought to compress the network while maintaining high performance. Network pruning reduces the cost of resources by eliminating redundant components from the network. It can be divided into two types, namely, unstructured and structured pruning. The former compresses the network using weight-level consideration. To eliminate unnecessary elements, weights that have little effect on the results are removed. Han et al. [13,14] pruned weights with small magnitudes. Dong et al. [15] evaluated the influence of weights based on changes in weight loss. Instead of using the weights of a well-learned network, an optimal network can automatically be identified by learning a model for compression [16]. These methods can save several parameters without reducing performance. However, special hardware is required to reduce computational costs and network parameters. Notably, structured pruning removes channel-level elements that have less effect on performance. Similar to unstructured pruning, a method to prune channels without affecting the results exists. Several methods determine and eliminate meaningless filters based on the L1 or L2 norm [17,18]. Another approach uses a geometric median to reduce redundant channels [19]. Another study directly learned the structure of a compressed network [20,21,22], allowing computational complexity to be reduced directly by reconstructing the network. These methods are highly compatible, as they can be applied to well-designed models. In particular, structured pruning does not require special hardware, and as such can be used for devices with different resource budgets. However, most pruning methods only deal with cutting out redundant components, and do not consider the reconstruction process.

This study proposes a lossless reconstruction method for channel-based pruning. Most recent convolutional neural networks (CNNs) are composed of residual blocks, skip connections, and convolution layers [23,24,25]. We reconstructed the pruned model considering these components. In order to design a deeper model, a residual block is used to avoid a vanishing gradient through identity mapping. As the spatial information of the feature maps can be damaged during the reconstruction process, a union operation was applied to preserve spatial information while reconstructing the network. A skip connection was used to alleviate information loss by transferring low-level features to a deeper layer. After the pruning process, only a few feature maps containing important information remained. Index alignment was then used appropriately to transfer the remaining feature maps. We considered batch normalization to reconstruct the convolution layer without performance degradation. Batch normalization directly affects the output of the convolution layers, and as such ought to be handled simultaneously.

In our experiments, we applied the proposed reconstruction method after applying the existing channel-based pruning method. The results show that the proposed method effectively reduces both computational complexity and the number of parameters without impacting performance. Experiments on downstream tasks, such as image classification, object detection, and semantic segmentation, were conducted in order to demonstrate the compatibility of our method. We observed that performing compression on a large model yields better results than compressing a well-designed small model. Moreover, the inference speed can be accelerated while maintaining performance on various devices. This indicates that the training time could be significantly reduced by changing the sequence of the compression process. The contributions of this study are as follows:

- We propose a lossless reconstruction method using a residual block, skip connection, and batch normalization layers after the pruning algorithm to remove redundant channels.
- We show that compressing a large model leads to better performance than compressing a well-designed small network.
- We demonstrate that our method is able to significantly reduce runtime by measuring the latency on Raspberry Pi and Jetson Nano.

The rest of the paper is organized as follows: Section 3 introduces our lossless reconstruction method; validation of various computer vision tasks, such as image classification, semantic segmentation, and object detection on various devices, are presented in Section 4; Section 5 presents a discussion; finally, Section 6 concludes the study.

## 2. Related Works

### 2.1. Architecture Search

CNNs have achieved considerable results in computer vision tasks; however, they require a large resource budget to ensure performance. To alleviate this trade off, research efforts have sought to design efficient models while preserving high performance. MobileNet [26] decomposes conventional convolution into depth-wise and point-wise convolution. This effectively approximates convolution, and significantly reduces the amount of computation required. EfficientNet [23] categorizes the parts that affect the cost of the network into depth, width, and resolution. Based on these components, it determines the optimal combination for a network with high performance and good efficiency. Unlike these methods, which require human effort, Neural Architecture Search (NAS) [27] discovers the optimal network structure according to the computation cost by applying reinforcement learning. To reduce the search time, DARTS [28] effectively reduces the processing time required to identify an architecture by proposing a differentiable search method. Rather than discovering the architecture directly from large search spaces, RegNet [29] designs search spaces and discovers the network structure. However, these methods are inflexible to the resource requirements of the target device, as they require considerable effort and cost to determine the optimal network.

### 2.2. Unstructured Pruning

Studies have been conducted on compressing a designed network in order to better correspond to the resource budget of the target device. Network pruning lowers the computational cost of a network by eliminating insignificant elements. This process can be categorized into unstructured or structured pruning. The former eliminates meaningless and redundant weights. Several studies have proposed pruning methods that disconnect unimportant connections [30,31]. Han et al. [13,14] pruned networks based on the magnitude of the weights and applied a quantization process for further compression. Net-trim [32] compresses networks by minimizing errors through special convex optimization. However, this method does not achieve a high compression rate. Further, Dong et al. [15] reduced the less meaningful weights based on the second derivative of the loss function. Hu et al. [33] eliminated insignificant weights by analyzing the output of a large dataset. Molchanov et al. [34] sparsified fully connected and convolution layers using variational dropout. To automatically reduce network size, AutoPrune [16] is a pruning method with recoverability that is able to learn the thresholding function and prune the model simultaneously. Lee and Hwang [35] proposed layer-wise compression by modeling the weight distribution of each layer using a Gaussian mixture model. Yu et al. [36] solved the combinatorial extension of (OBS) [31] using tractable formulation. A strong bias was induced when pruning was performed before or during training. Wimmer et al. [37] introduced interspace pruning to deal with this bias. Although unstructured pruning achieves a high compression rate, it requires special hardware and data reconstruction methods, such as a hash map, to reduce the use of actual resources.

### 2.3. Structured Pruning

Another approach, structured pruning, eliminates less meaningful components from the channel dimensions. By directly pruning structured elements, the required computation and number of parameters can be reduced without specialized hardware or extra costs. A number of pruning methods based on the channel-wise L1 and L2 norms are similar to unstructured pruning [17,18]. aPoZ [33] identifies unimportant values by considering the zero rate after the activation function. To address the redundancy of each layer, He et al. [19] proposed using a geometric median-based pruning method rather than the L1 or L2 norm. ThiNet [38] prunes the filters of the current layer using the output feature map of the layer. NISP [39] obtains importance scores by considering the final response layer and formulates network pruning as a binary integer optimization.

Studies have been conducted to determine the optimal number of channels in each layer for achieving the best performance. To discover an appropriate structure, a hypernetwork is used to produce the weights and channels of the network [20,40]. LeGR [21] learns the global ranking of models using an evolutionary algorithm. To ensure high accuracy, Gao et al. [22] trained a performance prediction network to guide the pruning process. Li et al. [41] determined channel configuration using a random search. Shen et al. [42] pruned channels globally based on magnitude and gradient criteria. Unlike pruning-only methods, Hou et al. [43] proposed a pruning-and-regrowing method to avoid removing important channels. These methods can compress a network while ensuring high performance. However, they do not consider the potential performance degradation that can occur during the actual reconstruction process.

## 3. Methods

**Figure 1:**
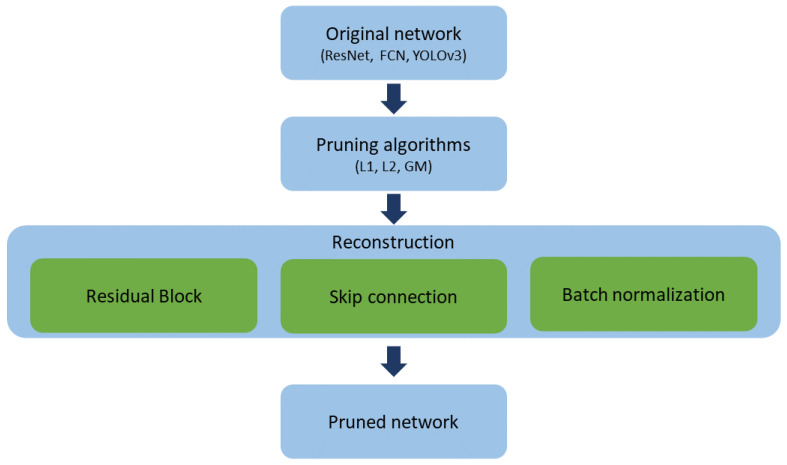
Pruning process with reconstruction.

Existing channel-based pruning methods are able to compress networks while achieving high performance. However, the reconstruction process has not been considered previously. This can cause performance degradation when the network is applied to a target device. This study proposes a reconstruction process to reduce the actual computation and number of parameters required without affecting the accuracy. To preserve performance, we focus on the residual block, skip connection, and convolution layer, which are the basic components of a CNN. Our overall pruning process is shown in Figure 1.

### 3.1. Residual Block

**Figure 2:**
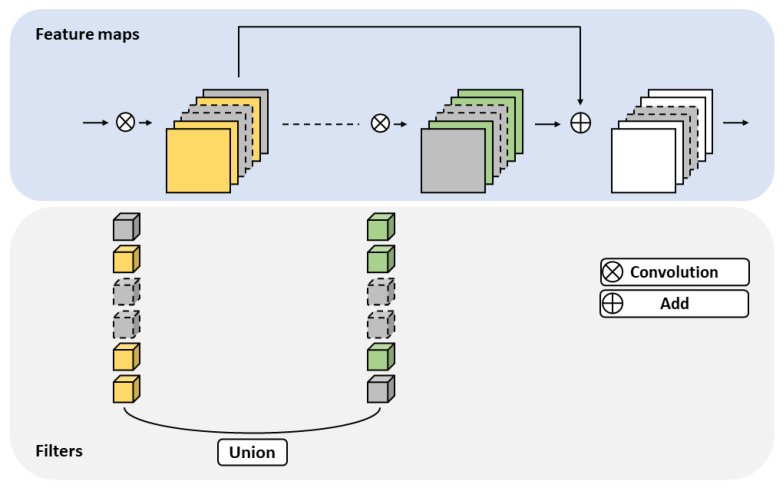
Overview of reconstruction method for residual blocks. The blue background represents feature maps of the residual blocks and the gray background represents the filters of the residual blocks. Each zero-value filter or feature map is represented in gray. Yellow and green filters represent the filters remaining after pruning. Yellow and green filters are used during union operations. Dashed items are those removed after pruning.

A residual block was introduced into ResNet [2] in order to design a deeper network consisting of bottleneck layers and shortcut connections. Using the latter, we addressed the vanishing gradient problem by passing the loss along directly. Moreover, fast convergence was possible based on residual learning. Therefore, shortcut connections needed to be considered in order to ensure performance. After the pruning process, feature maps with zero values were produced by eliminating less meaningful filters, as shown in Figure 2.

As the channel-wise importance of the two layers to be added through the shortcut connection was not the same, filters with different indices were removed. If channels with zero value are cut out directly, feature maps with different indices must be added together, resulting in significant loss. As shown in Figure 2, we instead apply a union operation for index matching. To minimize performance degradation, the corresponding index should not be removed from another layer when a specific channel index is retained from one channel. Adopting a union operation guarantees this property, functioning as a type of padding and matching the indexes of both layers, allowing lossless reconstruction.

### 3.2. Skip Connection

**Figure 3:**
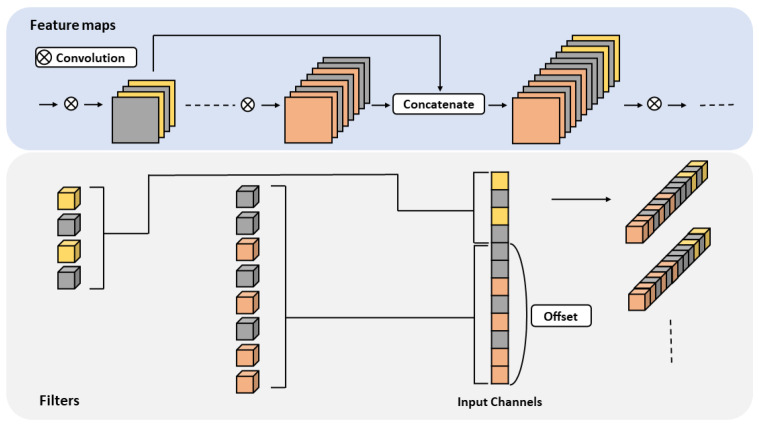
Overview of proposed reconstruction method for the skip connection. Gray indicates a value of zero, while yellow and red represent the remaining filters in the shallower and deeper layers, respectively. Our method offsets the remaining index of a shallow layer as a filter size of a deeper layer during the reconstruction stage. Finally, the gray items are eliminated after pruning.

A skip connection transfers low-level features to deeper layers. It is widely used to improve representation power because it aggregates detailed information in the shallow layer and global features in the deeper layer. A skip connection concatenates low and high-level feature maps to transfer information. As shown in Figure 3, the less important filters are set to zero values during the pruning process, and the feature maps corresponding to these filter indices have zero values.

Because features with zero values are meaningless when operating on concatenated feature maps, the filter corresponding to the zero-valued feature maps can be removed as well. Index alignment is required to accurately remove filters with redundant computation. As shown in Figure 3, lower-level feature maps are shifted by the number of channels of higher-level feature maps when concatenating through the skip connection. For index alignment, the offset is assigned to low-level feature maps to the extent of the shift. Because the index corresponding to the zero value can be accurately removed, usage of the actual resource is reduced without performance loss.

### 3.3. Batch Normalization

**Figure 4:**
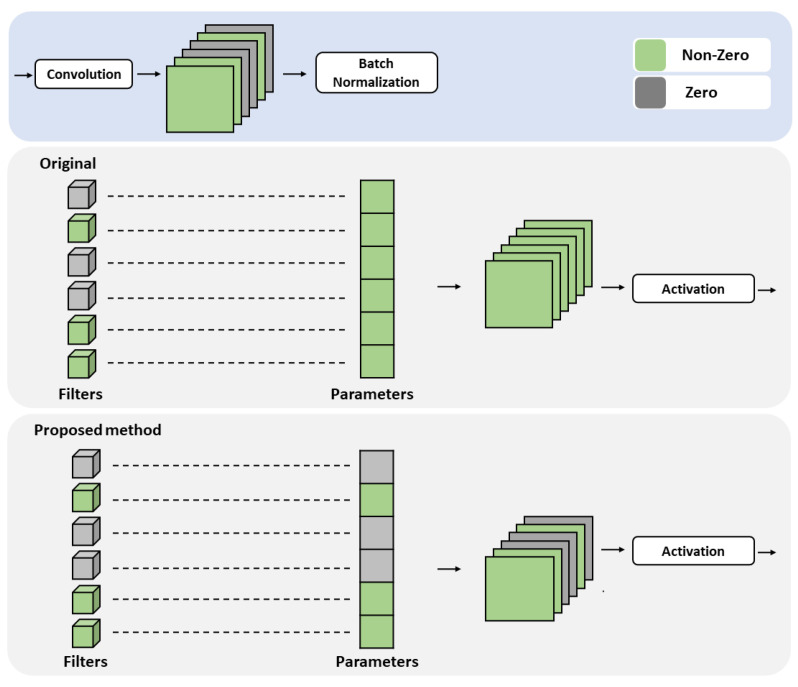
Comparison of different methods for dealing with the batch normalization parameters. Gray and green indicate zero and non-zero values, respectively. The proposed method prunes the batch normalization parameters to correspond with the pruned filters in the convolution layer.

Typically, the convolution layer consists of convolution, activation, and batch normalization. Most pruning methods use the weight of a convolution to determine its importance and then eliminate the less significant components. However, dealing only with convolution can cause performance degradation owing to activation or batch normalization. In particular, batch normalization [44] can solve internal covariance shifts, and has a significant effect on the network:

(1) $$\begin{array}{c} \mu_{B}=\frac{1}{m}\sum_{i=1}^{m}x_{i},\sigma_{B}^{2}=\frac{1}{m}\sum_{i=1}^{m}{\left(x_{i}-\mu_{B}\right)}^{2},\\ \hat{x_{i}}=\frac{x_{i}-\mu_{B}}{\sqrt{\sigma_{B}^{2}+\epsilon }},y_{i}=\gamma \hat{x_{i}}+\beta , \end{array}$$

where $B=\{x_{1},x_{2},...,x_{m}\}$ indicates a mini-batch, $x_{i}$ is the *i*th data in the mini-batch, and γ and β are the scale and shift, respectively, both of which are trainable parameters. Unlike the activation function, batch normalization affects the shift and scale transformation of feature maps. Therefore, it is sensitive to network pruning. Figure 4 shows the effects of batch normalization. After the pruning process, the less meaningful filters are removed, and the value of the corresponding feature maps should be zero. However, when batch normalization is not considered, these feature maps have non-zero values owing to scale and shift transformations. Because they affect the output of the network, information loss occurs if they are eliminated during the reconstruction process. To address this issue, we modified the pruning process by considering batch normalization. As shown in Figure 4, the proposed method eliminates both unnecessary filters and the corresponding parameters used for batch normalization in the pruning process. The feature maps retain values of zero because the scale and shift transformations have no effect. This approach to pruning enables network compression during the reconstruction process without any information loss.

## 4. Experiments

We experimentally demonstrated the effectiveness of the proposed lossless reconstruction method. First, we applied our proposed method to image classification, object detection, and semantic segmentation in order to show its compatibility for downstream tasks. Second, we measured the latency and FLOPs (FLoating point OPerations) on several devices to verify the actual compression. In an ablation study, we show that each of the proposed algorithms is effective for lossless reconstruction. Table 1 lists the hyperparameters used in the experiments.

### 4.1. Image Classification

We applied our method to ResNet [2], an image classification network. A residual block was used to address the vanishing gradient problem that occurs when designing deeper networks. As the depth of the network increases, the performance improves and the computation becomes heavier. By controlling depth, networks with different costs can be designed for application to various devices.

In this experiment, we use ResNet101, ResNet50, ResNet34, and ResNet18. ResNet101 and ResNet50 are designed with 101 and 50 bottleneck blocks, respectively, comprising a combination of 1 × 1 filters in the first case and a 3 × 3 filter in the second. By constrast, both ResNet34 and ResNet18 consist of basic blocks of 3 × 3 filters. For training, the networks use pre-trained models provided by Pytorch. We used SGD (Stochastic Gradient Descent) with a mini-batch size of 128 running on two GeForce 2080 Ti GPUs. The model was fine-tuned over 100 epochs starting with a learning rate of 0.01, then dividing it by five at 50 and 70 epochs. The momentum and weight decay were set to 0.9 and 0.0001, respectively. For compression, L1, L2 [17,18], and geometric median (GM)-based [19] pruning methods were used. To evaluate the accuracy of the networks, we used the CIFAR10 dataset (https://www.cs.toronto.edu/~kriz/learning-features-2009-TR.pdf) (accessed on 30 December 2022) [45], which consists of 50k/10k training and testing images, respectively, with ten classes. As presented in Table 2, “Acc.↓(%)” is the difference from the baseline for ResNet101.

**Figure 5:**
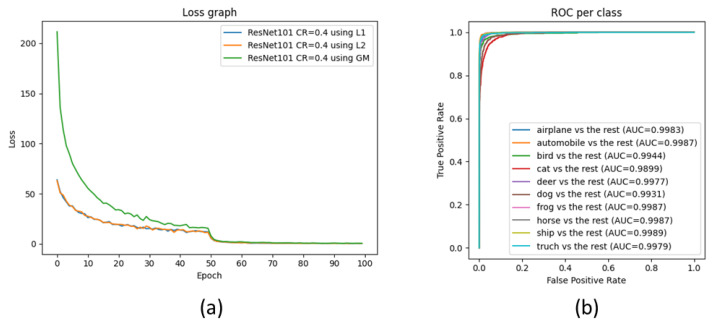
(**a**) Training loss for ResNet101 pruned at 40% using L1, L2, and GM methods and (**b**) the ROC curve per class for the best ResNet101 pruned by the L1 method.

First, we compare ResNet50 with the compressed ResNet101 comprising bottleneck blocks. In order to conduct a fair comparison, we compressed ResNet101 by 40% to achieve FLOPs comparable to those of ResNet50. Table 2 shows that the actual resources are reduced by the proposed reconstruction process. With similar costs, the accuracy loss of the compressed network is 0.04%, whereas the smaller model has a performance gap of 0.63% compared with the baseline. The training loss and ROC curve of the pruned ResNet101 are shown in Figure 5. For a fair comparison with ResNet34, which consists of basic blocks, ResNet101 and ResNet50 were compressed with compression rates of 0.45 and 0.1, respectively. The results show that the networks compressed with ResNet101 and ResNet50 have performance losses of 0.14% and 0.15% from baseline, respectively, whereas the one compressed with ResNet34 has an accuracy difference of 0.83%. Compressing ResNet50 increases the performance. This shows that network pruning has a dropout effect and can help with generalization. Finally, we compare the smallest network, ResNet18, with the other compressed networks. To obtain the same resources, ResNet101, ResNet50, and ResNet34 were compressed to 0.7, 0.5, and 0.5, respectively. Table 2 shows that ResNet18 has a performance gap of 2.53% from the larger model, whereas the other networks have less performance loss.

This experiment shows that compressing a large model produces better performance than using a small network to reduce the computational cost and number of parameters. Moreover, the performance gap between the large and compressed models is not significant, indicating that the pruning method can remove only redundant channels while maintaining the capacity of the model. However, in the case of GM-based compression, performance degradation increases as the compression rate increases. This shows that the number of redundant channels is small and that meaningful channels can be removed at high compression rates. As actual resources can be reduced using the proposed method, they can be adaptively applied to the target device.

### 4.2. Semantic Segmentation

Our method was applied to semantic segmentation by compressing a Fully Convolutional Network (FCN) [46] from Pytorch. The spatial information used to performs pixel-wise classification is crucial for image segmentation. The FCN replaces fully connected layers, which cause deterioration of spatial information, with convolutional layers. This network is divided into feature extraction using residual blocks and upsampling for pixel-wise classification. In order to preserve the spatial information, we apply our method only to feature extraction.

To train the large network, we used the Pascal VOC dataset (http://host.robots.ox.ac.uk/pascal/VOC/) (accessed on 30 December 2022) [47], which has twenty classes and 11,530 training/validation images. Starting with pre-trained FCN models provided by Pytorch, we fine-tuned the models for 100 epochs using the per-pixel multinomial logistic loss and validated them with the standard metric of mean pixel intersection over union. We used an SGD with a mini-batch size of 16 running on a GeForce 2080 Ti GPU. The model started with a learning rate of 0.01, which was divided it by five at 60 and 80 epochs. The weight decay and momentum were set as 0.0001 and 0.9, respectively. To evaluate the network performance, we used two metrics, namely, the global accuracy and mean intersection over union (mIoU), defined in the FCN [46] as follows:

(2) $$G_{Acc}=\frac{\sum_{i}n_{ii}}{\sum_{i}t_{i}},$$

(3) $$mIoU=\frac{\left(1/n_{cl}\right)\sum_{i}n_{ii}}{\left(t_{i}+\sum_{j}n_{ji}-n_{ii}\right)}$$

where $n_{ij}$ is the number of pixels of class *i* predicted to belong to class *j*, $t_{i}$ = $\sum_{j}n_{ij}$ is the total number of pixels of class *i*, and $n_{cl}$ is the number of classes.

We compressed a large model, FCN-ResNet101, to show the effectiveness of the proposed reconstruction. L1, L2, and GM-based pruning methods were used, and the compression rate was set to 0.3 in order to achieve similar FLOPs with FCN-ResNet50. For comparison, FCN-ResNet50, a smaller model with low resource usage, was trained using the same settings as the large model. Table 3 shows that the FLOPS and number of parameters decrease when applying our method. The model performs the best when compressed with L2-based pruning, with a performance loss of 0.64 for $G_{A}cc$ and 1.9 for mIoU. The results show that the pruning networks based on L1 and L2 have similar $G_{A}cc$ and mIoU results, while the GM-based method performs significantly worse. As the methods based on L1 and L2 cut out channels with small norms, the loss of spatial information is small. However, because GM-based pruning compresses the network based on redundancy, the norms of the removed channels can be considerable. This causes a relatively large loss of spatial information compared to pruning based on L1 and L2. Next, we compare the well-designed small model and the compressed network. With the exception of the GM-based method, Table 3 shows that the compressed network achieves higher $G_{Acc}$ and mIoU with similar resources as the smaller FCN-ResNet50 model. In the small model, the feature extractor consists of ResNet50, which has a shallower depth than the large model. Therefore, the capacity of the network is small. These result show that compressing a large model with high capacity can result in perform better than using a well-designed smaller network with low capacity.

### 4.3. Object Detection

We applied the proposed method to YOLOv3 [48], which is a single-stage object detection network. To compress the network, we implemented our reconstruction method on the Darknet-53 feature extractor. Darknet-53, proposed in YOLO [6], is faster and has better performance compared to earlier backbone networks such as ResNet. Because our reconstruction method consists of residual blocks, skip connections, and batch normalization, it can be applied effectively in this scenario. For training and evaluation, we used the Pascal Visual Object Classes dataset [47], which comprises 11,000 images and 27,000 annotated objects in twenty classes. To measure performance, we used the mean average precision (mAP), defined as follows:

(4) $$\begin{array}{c} Recalls=\frac{TP}{\left(TP+FN\right)},Precisions=\frac{TP}{\left(TP+FP\right)}, \end{array}$$

(5) $$\begin{array}{c} AP=\sum_{k=0}^{k=n-1}\left[Recalls\left(k\right)-Recalls\left(k+1\right)\right]\cdot Precisions\left(k\right) \end{array}$$

mAP is calculated using a graph of the Precision–Recall (PR) curve based on the threshold. Using this curve, precision and recall are obtained by the confusion matrix, which consists of the True Positives (TP), True Negatives (TN), False Positives (FP), and False Negatives (FN) between the precision class and actual class relationship. Recall and precision are represented using Equation (4) depending on the IoU threshold. Using this feature, we produce the PR curve for the AP using Equation (5), where *n* is the number of thresholds; then, the average AP for each class is used to obtain the mAP.

Although YOLOv3 is significantly less expensive than other object detection networks, it nonetheless incurs high costs when used in resource-limited devices. For use with such devices, YOLOv3-tiny was designed with a shallower structure than the original model. We compressed the large model and compared it with the YOLOv3-tiny model. To train YOLOv3-tiny, we used the same setting as for the original model. The compression rate was selected as 90% in order to ensure similar FLOPs as YOLOv3-tiny, and the pruned model was retrained over 100 epochs. In these experiments, we adopted the L1, L2, and GM-based pruning methods to remove unnecessary channels.

Table 4 shows the resources and mAP of the compressed network and the YOLOv3-tiny model. After applying our method, the actual FLOPs and parameters of the network are significantly reduced, resulting in performance degradation of about 0.12. Unlike in semantic segmentation, there is little performance difference between the pruning methods. Compared with YOLOv3-tiny, the compressed network shows higher performance with fewer resources. YOLOv3-tiny is designed as a shallow network to reduce costs, and has a small capacity. However, a network that compresses a large model through pruning methods maintains a high-capacity configuration and removes only redundant components. Therefore, compressed networks can achieve better performance than well-designed small models, as shown in Table 4.

### 4.4. Latency

There are many downstream tasks in which the run time is critical. For instance, in autonomous driving, information must be processed in real-time using a deep learning network. High-resource devices are required to ensure real-time performance. However, these devices are usually expensive, and cannot be applied to services or products. Instead of using these high-cost devices, a real-time task has been adopted on low-cost devices using efficient methods [49]. To demonstrate the application of real-time operation on both low-end and high-end devices, we measured the inference time after compressing the network.

In these experiments, YOLOv3, which is a real-time object detection network, was used as the large model. We compressed the network to 50%, 70%, and 90%, then compared it to YOLOv3-tiny. The networks were applied to Raspberry Pi4 and Jetson Nano, both of which are low-cost devices, and an RTX 2080 Ti GPU, which is a high-end device. While the Raspberry Pi 4 uses a quad-core ARM CPU, it does not have CUDA cores to support parallel operation of multiple neural networks for applications such as image classification, object detection, and segmentation. The Jetson Nano, another low-cost device, has CUDA cores and a quad-core ARM CPU. Therefore, it can process faster than Raspberry Pi 4.

Table 5 shows that real-time operation is possible for all models on the RTX 2080 Ti. On the Jetson Nano, the large model has a latency of 0.5347, while on the Raspberry Pi 4 it has a large inference time of 5.0755. After the network is compressed using the proposed reconstruction method, the actual latency tends to decrease as the compression rate increases. Despite being applied to low-resource devices, the run times of the 90% compressed model are 0.1009 and 0.6227 on Jetson Nano and Raspberry Pi 4, respectively. Moreover, we compared a well-designed small network with the compressed network. As shown in Table 5, the compressed network shows better performance than the smaller YOLOv3-tiny network. However, the latency is slightly higher, at 0.0203 for the Raspberry Pi 4 and 0.0188 for the Jetson Nano. Due to preserving their depth-based structure, compressed models require more time to read and write to memory. These results show that the actual run time can be reduced by reconstructing the compressed network using our method. Moreover, the network compressed using our proposed method has higher performance than a well-designed small network on all of the devices used in the experiments.

Additionally, we compared our method with a previous work [40] on the CIFAR10 dataset. The results shown in Table 6 demonstrate that our reconstruction process can accelerate pruned models. Furthermore, while maintaining similar latency, the pruned model using the L2 method shows better improvement in terms of the Top-1 Error (%) compared to the DHP-38.

### 4.5. Ablation Study

In this section, we describe various experiments conducted to demonstrate the influence of the proposed method on lossless reconstruction. First, we present the effectiveness of the union operation applied to the residual block. Then, we demonstrate that pruning with consideration batch normalization allows for reconstruction without performance loss. ResNet50 with the CIFAR10 dataset was used as the baseline for the experiment, and the training settings were the same as thosedescribed in Section 4.1. To measure performance, we evaluated fine-tuning accuracy by retraining after compression and comparing the accuracy results before and after reconstruction. This experiment shows that efficient training can be realized by applying the proposed reconstruction method to the pruning process.

#### 4.5.1. Applying Union Operation in Residual Block

Significant loss can occur when filter indices are different during this addition; thus, the indices of the filters that produce these two feature maps should be matched. Two operations can be used, namely, union and intersection. Table 7 shows the results of the compressed model with a 0.5 compression rate when applying each operation. Here, “OR” and “AND” respectively indicate the union and intersection operations. The results show that the intersection operation causes significant accuracy loss between the fine-tuning accuracy and reconstruction accuracy, whereas the union operation has a lossless reconstruction result. For the intersection operation, the filter is not removed and the filters of the two layers remain. Notably, information can be lost because the channels in the two layers do not have the same importance in the pruning process. However, in the union operation, even if only one of the filters remains in the deeper or shallow layer, the filter is not removed. As this operation maintains all important channels in both layers, information is preserved and lossless reconstruction is possible when parameters are removed.

#### 4.5.2. Influence of Pruning Batch Normalization Layer

In Section 3.3, we described an experiment examining the impact of reconstruction considering batch normalization. For this experiment, the network was compressed at a compression rate of 0.5, then the pruning results with and without considering batch normalization were compared. As shown in Table 8, the fine-tuning accuracies of both networks are nearly identical. However, in the case of pruning that does not consider batch normalization, performance degradation occurs after reconstruction. The scale and shift transformations of batch normalization affect the output of the network, because they can produce non-zero feature maps. When filters are removed during the reconstruction process, non-zero feature maps are removed as well, resulting in information loss and performance degradation. However, when pruning is performed considering batch normalization, the feature maps of the pruned channel maintain values of zero. Because no information is lost by removing them, the performance is maintained after the reconstruction process.

#### 4.5.3. Influence of Compression Process

**Figure 6:**
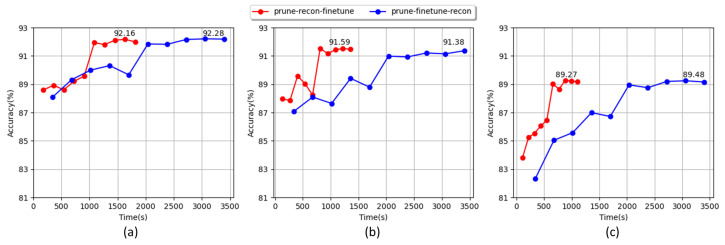
Comparison of training time required during the pruning–reconstruction–fine tuning process (red line) and pruning–fine tuning–reconstruction process (blue line) using pruned ResNet34 on the CIFAR-10 database; (**a**–**c**) refer to models with compression rates of 0.4, 0.6, and 0.8, respectively. The training time and accuracy were recorded over ten epochs. The data labels show the best accuracy of each model.

The pruning process is divided into two steps, namely, removing less important components and retraining to recover performance. However, because the actual reconstruction is not performed, the time consumption in the retraining step is significant. To address this issue, we prune the channels and retrain them following the proposed reconstruction method. For the experiment, ResNet34 with the CIFAR10 dataset is used as a baseline. The networks were compressed with compression rates of 0.4, 0.6, and 0.8 and retrained over 100 epochs. In Figure 6, the blue line represents the result of applying the reconstruction process after retraining and the red line represents the result of applying the proposed reconstruction before retraining. The results show that the best performance is similar for all compression rates. However, the time consumption in the retraining step is significantly reduced, especially at the compression rate of 0.8, with time savings of about three times the required time. This experiment shows that the proposed method can effectively reduce the required time by accelerating the network training time.

## 5. Discussion

In this study, we have introduced a method for lossless reconstruction after pruning to reduce practical run time. We have demonstrated the effectiveness of our method by measuring the resulting latency on several devices. Our experimental results demonstrate that our method is important for acceleration when researching pruning criteria.

### 5.1. Strengths

Previous studies on structured pruning have focused on determining redundant filters. However, here we address the reconstruction of networks to eliminate meaningless components of the network and accelerate the latency, which is shown to be effective on both the Raspberry Pi and Jetson Nano.

### 5.2. Weaknesses and Limitations

Because our method is conducted following the pruning process, the resulting acceleration in terms of latency depends on the heuristic compression ratio. Therefore, an appropriate compression rate needs to be chosen in order to significantly reduce latency. Our approach results in pruned networks composed of convolution layers, skip connections, and residual blocks without any loss. Although there are many other types of modules used in networks, these limitations do not apply to them.

## 6. Conclusions

In this study, we propose a lossless reconstruction process to reduce actual network resource usage. We used residual blocks, skip connections, and convolution layers, which are the usual basic components of CNNs, to compress the network. We adopted the union operation to deal with shortcut connections in the residual blocks and used index alignment to reconstruct the skip connections. Moreover, batch normalization was considered to prune the convolution layer. In our experiments, we show that the proposed method is compatible with various downstream tasks by applying it to ResNet, FCN, and YOLOv3. Our results show that compressing a large model produces better results than a well-designed small model with the same resources. In addition, we measured the actual latency on various devices to show that real-time operation is possible even on resource-limited devices. Through an ablation study, we validated the consideration of each element for reconstructing the network without performance degradation. Future works should focus on pruning networks while considering reconstruction for other tasks, such as face recognition and pose estimation, which require fast inference times. In addition to reducing the time needed to fine-tune pruned networks, it should be possible to decrease the entire training time, including when training large networks, to achieve compressed networks.

## Figures and Tables

**Table 1:** Hyperparameters of original and pruned models.

	Image Classification		Semantic Segmentation		Object Detection	
	**Original Model**	**Pruned Model**	**Original Model**	**Pruned Model**	**Original Model**	**Pruned Model**
batch size	128	128	16	16	16	16
GPU	2	2	1	1	1	1
epoch	200	100	100	50	150	100
optimizer	SGD	SGD	SGD	SGD	SGD	SGD
momentum	0.9	0.9	0.9	0.9	0.9	0.9
weight decay	0.0001	0.0001	0.0001	0.0001	0.0005	0.0005
initial lr	0.1	0.01	0.001	0.001	0.0001	0.0001
lr scheduler	divided by 10	divided by 5	divided by 5	divided by 5	cosine annealing	cosine annealing
	at 90, 140 epochs	at 50, 70 epochs	at 50, 70 epochs	at 25, 40 epochs	(0.0001∼0.00001)	(0.0001∼0.00001)

**Table 2:** Compression results of pruning ResNet on CIFAR-10. The “CR” column indicates the network compression ratio used for pruning, while “Method” denotes the network pruning criterion. In particular, the GM method denotes the criterion introduced in [19]. “Acc.↓ (%)” is the accuracy drop from the baseline model, with smaller values being better.

Model	CR	Method	FLOPs(M)	Params(M)	Acc. (%)	Acc.↓ (%)
ResNet101- baseline	-	-	1269.58	44.54	93.30	0.00
ResNet101	0.4	L1	621.86	21.86	93.26	**0.04**
	0.4	L2	621.78	21.89	93.20	0.10
	0.4	GM	633.40	23.39	93.06	0.24
ResNet50	-	-	661.00	25.54	92.67	0.63
ResNet101 - baseline	-	-	1269.58	44.54	93.30	0.00
ResNet101	0.45	L1	553.16	19.49	92.92	0.38
	0.45	L2	553.7	19.54	93.16	**0.14**
	0.45	GM	564.55	21.11	90.02	2.98
ResNet50	-	-	661.00	25.54	92.67	0.63
ResNet50	0.1	L1	571.66	22.17	93.15	0.15
	0.1	L2	571.62	22.16	93.11	0.19
	0.1	GM	573.54	22.45	93.11	0.19
ResNet34	-	-	585.31	21.78	92.47	0.83
ResNet101 - baseline	-	-	1269.58	44.54	93.30	0.00
ResNet101	0.7	L1	257.7	9.20	92.30	1.00
	0.7	L2	259.7	9.24	92.47	0.83
	0.7	GM	264.3	10.76	89.92	3.38
ResNet50	0.5	L1	267.48	10.48	92.61	0.69
	0.5	L2	269.02	10.47	92.70	**0.60**
	0.5	GM	275.22	11.88	92.52	0.78
ResNet34	-	-	585.31	21.78	92.47	0.83
ResNet34	0.5	L1	281.42	10.33	92.13	1.17
	0.5	L2	280.86	10.23	91.72	1.58
	0.5	GM	286.12	10.73	91.14	2.16
ResNet18	-	-	282.71	11.68	90.77	2.53

**Table 3:** Comparison results of pruning FCN on Pascal VOC. “$G_{Acc}$” indicates global accuracy; “$G_{Acc}$↓” and “mIoU ↓” are dropped from the baseline model, with smaller values being better; “CR” and “Method” have the same meaning as in Table 2.

Model	CR	Method	FLOPs(G)	Params(M)	$G_{\mathrm{Acc}}$	$G_{\mathrm{Acc}}$↓	mIoU	mIoU ↓
FCN_ResNet101 - baseline	-	-	397.86	54.31	94.87	0.00	75.1	0.00
FCN_ResNet101	0.3	L1	260.4	35.57	94.12	0.75	72.8	2.3
	0.3	L2	260.72	35.61	94.23	**0.64**	73.2	**1.9**
	0.3	GM	276.42	37.78	92.69	2.18	66.6	8.5
FCN_ResNet50	-	-	260.93	35.32	93.66	1.21	71.0	4.1

**Table 4:** Comparison of YOLOv3 on Pascal VOC. “mAP ↓” is mAP drop from baseline model, with smaller values being better; “CR” and “Method” have the same meaning as in Table 2.

Model	CR	Method	FLOPs (G)	Params (M)	mAP	mAP ↓
YOLOv3 - baseline	-	-	65.658	61.626	0.545	0.000
YOLOv3	0.9	L1	3.229	2.455	0.421	0.124
	0.9	L2	3.252	2.485	0.424	**0.121**
	0.9	GM	3.248	2.484	0.423	0.122
YOLOv3-tiny	-	-	5.518	8.714	0.370	0.175

**Table 5:** Latency comparison between YOLOv3 and YOLOv3-tiny on various devices. Here, “Latency” is calculated as the average processing time on CIFAR-10 evaluation images.

Device	Model	CR	mAP	FLOPs (G)	Latency (s)
Raspberry Pi 4 8GB	YOLOv3	-	0.545	65.658	5.0755
		0.5	0.543	14.671	1.5771
		0.7	0.536	10.019	1.1880
		0.9	0.423	3.248	0.6227
	YOLOv3-tiny	-	0.370	5.518	0.6024
Jetson Nano B01 4GB	YOLOv3	-	0.545	65.658	0.5347
		0.5	0.543	14.671	0.1647
		0.7	0.538	10.019	0.1236
		0.9	0.423	3.248	0.1009
	YOLOv3-tiny	-	0.370	5.518	0.0821
RTX 2080 Ti	YOLOv3	-	0.545	65.658	0.0076
		0.5	0.543	14.671	0.0030
		0.7	0.538	10.019	0.0024
		0.9	0.423	3.248	0.0016
	YOLOv3-tiny	-	0.370	5.518	0.0013

**Table 6:** Comparison of pruned ResNet50 with previously introduced pruned ResNet50 in DHP [40] running on CPU (Intel(R) Core(TM) i7-10700 CPU @ 2.90 GHz). “FLOPs Ratio (%)” is the ratio of FLOPs in a pruned model to a dense model. “Top-1 Error (%)” shows the dense model’s Top-1 Error (%), the pruned model’s Top-1 Error (%), and the difference between them. “Latency (ms)” has the same meaning as in Table 5. “Before reconstruction” refers to the latency when using the pruning process only, without reconstruction. “After reconstruction” refers to the latency after the use of our reconstruction process.

Model	Method	FLOPs Ratio (%)	Top-1 Error (%)	Latency (ms)	
				Before Reconstruction	After Reconstruction
ResNet50	L1	40.27	7.33 → 7.39 (+0.06)	8.647	5.467
	L2	40.50	7.33 → 7.30 **(−0.03)**		5.484
	GM	41.31	7.33 → 7.48 (+0.15)		5.676
	DHP-38 [40]	39.07	7.05 → 7.06 (+0.01)	5.333	

**Table 7:** Comparison between union and intersection operations applied to reconstruction. In the “Operation” column, “AND” means reconstructing residual blocks with the intersection operation, while “OR” means reconstructing residual blocks with the union operation. The “Finetune Acc” column denotes the accuracy after retraining the pruned model, “Reconstruction Acc” denotes the accuracy after reconstruction of the fine-tuned model, and “Acc Loss” indicates the accuracy drop between “Finetune Acc” and “Reconstruction Acc”, with smaller values being better.

Model	Method	CR	Operation	Finetune Acc	Reconstruction Acc	Acc Loss
ResNet50	L1	0.5	AND	92.82%	10.02%	82.80%
	L1	0.5	OR	92.82%	92.82%	0.00%
	L2	0.5	AND	92.70%	10.02%	82.68%
	L2	0.5	OR	92.70%	92.70%	0.00%
	GM	0.5	AND	92.80%	9.99%	82.81%
	GM	0.5	OR	92.80%	92.80%	0.00%

**Table 8:** Results of ResNet50 with a compression rate of 0.5 on the CIFAR10 dataset. In the “Batch norm” column, “✓” indicates pruning of the batch normalization layer corresponding with the convolution layer. The “Finetune Acc” column denotes accuracy after retraining the pruned model, while the “Finetune Acc”, “Reconstruction Acc”, and “Acc Loss” columns have the same meaning as in Table 7.

Model	Method	CR	Batch norm.	Finetune Acc	Reconstruction Acc	Acc Loss
ResNet50	L1	0.5	✓	92.53%	92.53%	0.00%
	L1	0.5		92.45%	89.32%	3.13%
	L2	0.5	✓	92.72%	92.72%	0.00%
	L2	0.5		92.71%	88.27%	4.44%
	GM	0.5	✓	92.42%	92.42%	0.00%
	GM	0.5		92.51%	55.17%	37.34%

## Data Availability

Not applicable.
